# Circulating Endothelial Cells as Promising Biomarkers in the Differential Diagnosis of Primary Angiitis of the Central Nervous System

**DOI:** 10.3389/fneur.2020.00205

**Published:** 2020-03-31

**Authors:** Milani Deb-Chatterji, Hans Otto Pinnschmidt, Yinghui Duan, Vivien Haeussler, Björn Rissiek, Christian Gerloff, Götz Thomalla, Tim Magnus

**Affiliations:** ^1^Department of Neurology, University Medical Center Hamburg-Eppendorf, Hamburg, Germany; ^2^Institute of Medical Biometry and Epidemiology, University Medical Center Hamburg-Eppendorf, Hamburg, Germany

**Keywords:** PACNS, vasculitis, biomarker, endothelial cells, stroke, autoimmune diseases

## Abstract

**Background:** Diagnosis of primary angiitis of the central nervous system (PACNS) and discrimination of PACNS from its mimics, e. g., reversible cerebral vasoconstriction syndrome (RCVS) or moyamoya disease (MMD) as non-inflammatory vasculopathies, still remain challenging. Circulating endothelial cells (CEC) are well-established markers for endothelial damage and potential biomarkers in PACNS. This study aimed to investigate if CECs may also help to distinguish an active PACNS from its important differentials (RCVS, MMD).

**Methods:** CECs were assessed in 47 subjects. Twenty-seven patients with PACNS were included, seven with an active disease (aPACNS), 20 in remission (rPACNS). Seven patients with RCVS/MMD were analyzed. Thirteen healthy subjects served as controls (HC). CECs were measured by immunomagnetic isolation from peripheral venous blood. Mann-Whitney-*U*-Tests were applied for between-group comparisons. The Benjamini-Hochberg-procedure was applied to adjust for multiple comparisons.

**Results:** In aPACNS, CECs were significantly elevated compared to HC (480 vs. 40 CEC/ml, *p* < 0.001) and rPACNS (54 CEC/ml, *p* < 0.001). RCVS/MMD patients showed higher CEC levels (288 CEC/ml) than HC (*p* < 0.001), but lower than those in aPACNS (*p* = 0.017). An adjustment for multiple comparisons confirmed prior significant differences. An increased CEC value (cut-off 294 CEC/ml) is indicative for an active PACNS [sensitivity 100%, 95% confidence interval (CI) 63–100%; specificity 93%, CI 81–98%].

**Conclusions:** CECs may serve as biomarkers for diagnosis, treatment monitoring, and also for differential diagnosis of PACNS. CECs seem to be a marker of endothelial injury with higher levels in inflammatory than non-inflammatory vasculopathies. Larger patient samples are required to corroborate these findings.

## Introduction

Primary angiitis of the central nervous system (PACNS) is an inflammatory disease affecting medium or small vessels of the CNS. PACNS is a rare condition (1) but remains an important differential diagnosis in stroke of young adults (2.2%) (2). Given that clinical manifestations show a broad, insidious and unspecific variety (3), and owing to the low specificity of most of the diagnostic procedures, diagnosis remains a challenge. At present, brain biopsy is the only eligible technique to establish a definite diagnosis of PACNS. However, even this gold-standard procedure is hallmarked by a low sensitivity (4–6). The potential aggressive course of the disease necessitates a high diagnostic accuracy for safely instituting the required immunosuppressive treatment. An accurate workup of PACNS mimics is needed. In particular, it is important to exclude other diseases which bear the closest resemblance to PACNS such as reversible cerebral vasoconstriction syndrome (RCVS) or moyamoya disease (MMD), both representing non-inflammatory vasculopathies (7–9). We have previously shown that circulating endothelial cells (CEC) could be a potential biomarker in PACNS (10, 11). CECs are well-known as a marker of endothelial damage (12, 13). Detachment of endothelial cells from the vessel wall either by mechanical injury, inflammatory or non-inflammatory endothelial damage is presumed to be the crucial pathophysiological mechanism (14). The endothelial cells are circulating in the blood and can easily be enumerated after isolation (15). Studies of small vessel vasculitis associated with antineutrophil cytoplasmatic antibodies (ANCA) showed increased CECs in patients with an active disease whereas CEC levels decreased under successful immunosuppressive treatment (16) indicating CECs being a diagnostic marker but also a marker of activity. In line with this, we demonstrated highly elevated CEC numbers also in patients with an active PACNS compared to patients in remission and healthy controls (10, 11). The present study aimed to verify these results and address the question if CECs may also serve as biomarkers to distinguish an active PACNS from its important differential diagnosis.

## Patients and Methods

### Patients and Healthy Controls

A total number of 47 individuals from the University Medical Center Hamburg-Eppendorf, Hamburg, Germany, were included in the study. Twenty-seven patients (*n* = 27) suffered from PACNS, whereas in 15 of them the diagnosis was biopsy-proven and 12 patients (*n* = 12) had a probable PACNS (see Supplementary Information: “Patients with probable PACNS”). Of note, all patients with a probable PACNS were classified to the medium vessel variant (MVV) of the disease with multiple irregular alternating stenosis or occlusions and dilatations in either conventional angiography or MR angiography (MRA) (17, 18). Patients with PACNS were allocated to two subgroups according to disease activity. Patients were considered to have an active course of disease (*n* = 7) when they were newly diagnosed or had a relapse. They all suffered from acute neurological symptoms, and in brain MRI new pathologies, e.g., ischemic stroke, were detected. Dark-blood-imaging (DBI) was performed to reveal an enhancement of the vessel walls, and an angiography (digital subtraction angiography, DSA, and/or MRA) was done to detect new or progressive vessel irregularities, both indicating an active MVV of the disease. CSF analysis was carried out in patients with disease onset to confirm an active inflammatory process of the CNS, e.g., a pleocytosis. Systemic inflammatory and infectious diseases were carefully ruled out in these patients by laboratory analyses (e.g., antinuclear and anti-neutrophil cytoplasmatic antibodies, human immunodeficiency virus, and others) and CSF analyses (e.g., varicella-zoster virus and others). CEC measurement was performed during this active course of disease. Patients with PACNS were considered to be in remission (*n* = 20) at time of blood sampling when they either had no clinical symptoms or were clinically stable under a successful immunosuppressive treatment. Brain imaging was performed to exclude any new or progressive pathological findings. Seven patients were included into the study after being diagnosed with RCVS (*n* = 4) or MMD (*n* = 3) (see Supplementary Information: “Patients with RCVS and MMD”). Blood sampling in RCVS was done when patients suffered from new, acute neurological symptoms (e.g., severe or thunderclap headache) with or without previous trigger factors, e.g., head trauma, and DSA revealed brain vessel irregularities. A cranial MRI was performed to detect any RCVS-associated cerebral pathologies, e.g., ischemic stroke. CSF analysis was done to exclude any inflammatory abnormalities, and an ultrasound of brain supplying vessels was performed to rule out a germane arteriosclerosis. All patients received follow up angiography 2–4 months after disease onset. All subjects with MMD had acute neurological symptoms, e.g., focal neurological deficits, at time of CEC assessment. Brain MRI was performed to reveal new pathologies, e.g., ischemic stroke or intracranial bleeding, and DBI was done to rule out any contrast-enhanced vessel walls. Ultrasound of brain supplying vessels was carried out to exclude a significant arteriosclerosis to which the vessel abnormalities can be secondary. Laboratory and CSF analyses were performed to rule out any inflammatory or infectious diseases. DSA exposed suspicious results for MMD according to the diagnostic criteria. The diagnosis of PACNS, RCVS, and MMD was performed interdisciplinary between a neurologist and neuroradiologist on a case-by-case basis. Thirteen healthy individuals (median age 30 years, 26.5–52.5) served as the control group. The local ethics committee (Hamburg, Germany; PV5340) approved the study. Written informed consent was obtained from all participants or proxies.

### CEC Measurement

**Figure 1:**
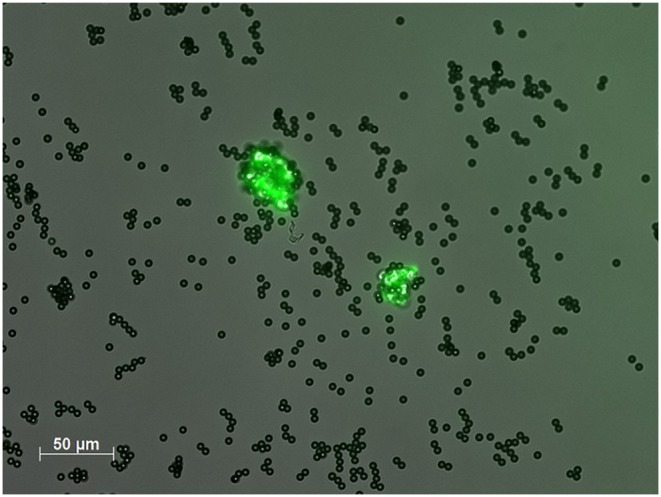
CECs in a patient with PACNS isolated from peripheral venous blood. Circulating endothelial cells (CECs) were isolated by CD146-coated dynabeads and a fluorescence-coated (FITC) lectin (*Ulex europaeus agglutinin 1*, UEA-1) in an endothelial cell specific double-staining method. CECs were enumerated in a counting chamber using a fluorescence microscope.

Peripheral venous blood was collected by atraumatic venipuncture. The first tube after blood sampling was discarded to minimize the risk of false-positive CEC counts. The blood was immediately processed at 4°C after withdrawal (see Supplementary Information: “Detailed information on CEC measurement”). In particular, CECs were assessed by immunomagnetic isolation as described in detail elsewhere (19). Briefly, blood (1ml) was blended with 1 ml buffer (phosphate buffered saline, 0.1% bovine serum albumin, and 0.6% sodium citrate) at 4°C. After adding 20 μl of the FcR blocking agent Octagam (Octapharma, Langenfeld, Germany) and 50 μl of the CD146 antibody (Biocytex, Marseille, France)—coated dynabeads (Invitrogen by Thermo Fisher Scientific, Pinneberg, Germany), the sample was incubated in a head-over-head-mixer for 30 min at 4°C. After adding the Fluorescein isothiocyanate (FITC)–labeled Ulex Europaeus agglutinin type 1 (UEA-1) solution (100 μl; 2 mg/ml, Sigma-Aldrich, Munich, Germany) the sample was incubated for 1 h at darkness at 4°C. The sample was washed three times with buffer before the cell-bead suspension was dissolved in 200 μl buffer. Cells were enumerated in a Nageotte counting chamber (Marienfeld Superior, Lauda-Königshofen, Germany) by using a fluorescence microscope (Figure 1).

### Statistical Analysis

Statistical analysis was performed by using SPSS (Version 23.0; IBM, Armonk, New York).

Results of Kolmogorov-Smirnov tests indicated a non-normal distribution of the CEC values in most of the patient groups. Levene test results indicated a high variance heterogeneity among the groups. Therefore, standard descriptive statistics were reported as median and interquartile ranges for CECs as a continuous variable. For between-group comparisons Mann-Whitney-*U*-tests were employed, assuming a target alpha level of 0.05. To control the false discovery rate (= 5%) of multiple testing the Benjamini-Hochberg procedure was applied. Categorical variables were expressed as percentages.

ROC analysis was performed to examine the diagnostic potential of CEC. To derive a cutoff value representing the maximum potential diagnostic effectiveness of CEC, the maximum value of the Youden index was determined, and the corresponding Clopper-Pearson confidence intervals for sensitivity and specificity were computed. All statistical tests were two-sided.

## Results

### Patients With an Active PACNS

Seven patients (*n* = 7) had an active disease course of PACNS (median age 48 years, 38–59 years, 71.4 % male) and had acute neurological symptoms (see Supplementary Tables: “Patients with active PACNS”). Three of them were diagnosed by histological evidence and four patients had a probable PACNS. Four patients suffered from disease onset, three had a relapse. These patients suffered from, e.g., new focal neurological deficits due to contrast-enhanced brain lesions, acute ischemic, or hemorrhagic stroke displayed on MRI. In all of these patients immunosuppressive therapy was either initiated, changed or planned to be initiated, or changed owing to this active disease episode.

### Patients With PACNS in Remission

Twenty patients (*n* = 20) were treated successfully with immunosuppressants and, thus, were in remission at time of CEC measurement (median age 50.5 years, 39–60 years, 50% male) (see Supplementary Tables: “Patients with PACNS in remission”). Eight patients had no clinical symptoms, whereas 12 patients were in a stabilized clinical condition with residual neurological symptoms. None of them showed clinical and/or imaging signs of disease activity. In 12 patients diagnosis had been made by brain biopsy, whereas eight patients had a probable PACNS (see Supplementary Information: “Patients with PACNS in remission”).

### Patients With RCVS and MMD

A total number of seven patients with a non-inflammatory vasculopathy (RCVS, MMD) were included in the study (median age 45 years, 29–51 years, 85.7% female) (see Supplementary Tables: “Patients with MMD and RCVS”). All patients with RCVS suffered from severe headache, whereas thunderclap headache (TCH) occurred in two of them. In the latter ones trigger factors (head trauma and postcoital) precipitated disease onset and the cranial MRI scans showed no abnormalities. Another patient had a severe peripartum headache with an unremarkable brain MRI. One patient showed minor SAH and ischemic stroke in MRI accompanied by a remarkable, bilateral “string and beads”-appearance of the cerebral vessels. Of note, this patient had a significant response of the vessel irregularities to an intra-arterial application of nimodipine. An aneurysm was carefully excluded by conventional angiography. Follow up imaging 2/4 months after symptom onset showed a persistent vessel abnormality in one patient, majorly improved vessel alterations in two cases, and completely resolved vascular changes in the other patient. The subjects with MMD suffered from an uni- or bilateral steno-occlusive process of the distal internal carotid artery (ICA) and MCA accompanied by a moyamoya syndrome. The cranial MRI revealed ischemic stroke and/or an intracranial bleeding in these patients. In all patients superficial temporal artery (STA)-middle cerebral artery (MCA)—bypass was either discussed or already carried out.

### CEC Counts

**Figure 2:**
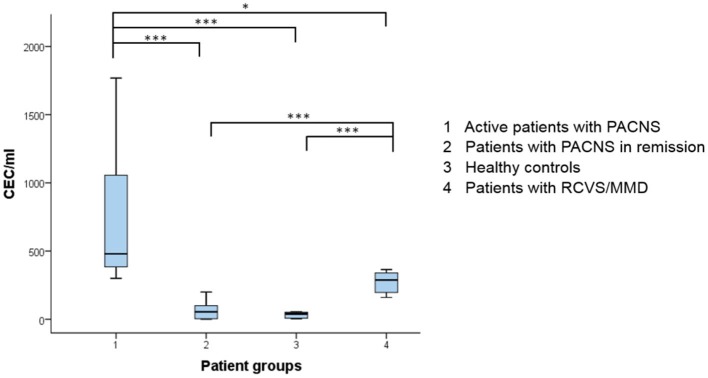
Distribution of CEC numbers in the present study population. Boxplot diagram showing the CEC values according to the group of patients included in the study. The stars reflect the level of significance. CEC, circulating endothelial cells; PACNS, primary angiitis of the central nervous system; RCVS, reversible cerebral vasoconstriction syndrome; MMD, moyamoya disease. *p < 0.05, ***p ≤ 0.001.

Patients with an active PACNS showed significantly higher CEC levels (480 CEC/ml, 304–1552) than PACNS patients in remission (54 CEC/ml, 4–100; *p* < 0.001, Figure 2) and also compared to healthy subjects (40 CEC/ml, 8–52; *p* < 0.001). In patients with RCVS/MMD, CECs were also significantly elevated (288 CEC/ml, 184–352) compared to healthy controls (*p* < 0.001), but they were still significantly lower when compared to patients with an active PACNS (*p* = 0.017). Patients with PACNS in remission showed lower CEC values than patients with RCVS/MMD (*p* < 0.001). We observed no differences between CECs in healthy individuals and patients in remission (*p* = 0.478).

Controlling the false discovery rate of multiple testing (six hypotheses) by the Benjamini-Hochberg procedure confirmed the prior significances in the between-group analyses (Table 1). In particular, CEC numbers between the active PACNS patients and PACNS in remission (*p* < 0.008), patients with RCVS and MMD (*p* < 0.042) as well as healthy controls (*p* < 0.025) were significantly different. In addition, patients with RCVS and MMD still showed a significant difference in CEC values compared to PACNS patients in remission (*p* < 0.017) and healthy controls (*p* < 0.033).

**Table 1:** Unadjusted *p*-values and Benjamini-Hochberg critical values for controlling false discovery rate (FDR = 5%).

**Patient groups**	***P*-values**	**Benjamini-Hochberg critical value**
aPACNS vs. rPACNS	0.000002*	0.008
rPACNS vs. RCVS/MMD	0.000009*	0.017
aPACNS vs. HC	0.000026*	0.025
RCVS/MMD vs. HC	0.000026*	0.033
aPACNS vs. RCVS/MMD	0.017*	0.042
rPACNS vs. HC	0.478	0.050

* *significant*.

*aPACNS, active PACNS patients; rPACNS, PACNS patients in remission; HC, healthy controls; RCVS, reversible cerebral vasoconstriction syndrome; MMD, moyamoya disease*.

Using the maximum value of the Youden index, a cutoff value of 294 CEC/ml was determined as a diagnostic criterion for differentiating between active PACNS patients as the case cohort and patients with PACNS in remission, healthy controls, and patients with RCVS/MMD as the reference cohort. With this cutoff value, the sensitivity of the test for the presence of active PACNS was 100% (95% confidence interval (CI) 63–100%), while the specificity was 93% (95% CI 81–98%).

## Discussion

PACNS is a rare inflammatory vascular disease confined to the CNS. Recently reported data compilations of PACNS patients have advanced the understanding of the disease (20, 21). However, the diagnosis remains a challenge owing to the low specificity of most of the diagnostic procedures. In addition, discriminating PACNS from its mimicking conditions is crucial due to the potential aggressive course of the disease and the need for appropriate treatment. New tools such as biomarkers, which facilitate the diagnostic workup, are required to establish and increase the certainty of the diagnosis. CECs have already been established as a sensitive and specific marker for endothelial injury (12). They are promising biomarkers in ANCA-associated small vessel vasculitis (15). CEC levels are increased in active vasculitis patients compared to healthy controls and patients under successful immunosuppressive treatment. Of note, we observed similar results in patients with PACNS (10, 11). We found highly elevated CEC numbers in patients with an active PACNS, whereas CECs were significantly lower in healthy subjects, patients in remission, but also in patients with cerebrovascular risk factors. These results indicate that CECs could not only be a diagnostic biomarker, but also monitor disease activity and treatment success. In the present study, we could reproduce and corroborate these findings. In a different patient population of another hospital (University Medical Center Hamburg-Eppendorf vs. Hannover Medical School) increased CEC levels were observed in patients with active PACNS compared to patients in remission and healthy controls.

Moreover, the relative elevation of CEC numbers between active patients and patients in remission/healthy controls, respectively, was similar between the study populations of the two different centers (present study sample: Δ 426/Δ 440 CEC/ml; previous study sample: Δ 368/Δ 372 CEC/ml, respectively) (10). In addition, this is the first study, which focused on discrimination of PACNS from its important differential diagnosis by CEC assessment. The non-inflammatory vasculopathies, RCVS, and MMD, are the most important differential diagnosis of PACNS. Misdiagnosis is common owing to overlapping imaging features. We demonstrate significantly higher CEC numbers in patients with an active PACNS compared to patients with RCVS/MMD, whereas CEC values of RCVS/MMD patients were higher than in healthy controls and patients with PACNS in remission. Of note, similar findings were observed in patients with MMD showing increased CEC levels compared to healthy controls (22).

Notably, our results were invariant after adjustment for multiple comparisons. In addition, we identified high sensitivity and specificity rates for CEC values in patients with active PACNS. Similar high results were assessed in patients with systemic ANCA-associated small vessel vasculitis (16). CEC counts were also shown to be elevated in other non-inflammatory vasculopathies, such as acute coronary syndrome (13) or stroke (23). In particular, Woywodt el al. demonstrated increased CEC values in patients with ischemic stroke compared to healthy controls and patients with cerebrovascular risk factors (23). However, when compared to patients with active PACNS these stroke patients showed significant lower CEC levels in the blood (10). These results were consistent to the findings gained in the present study. Detachment of endothelial cells from the vessel wall by endothelial injury seems to be the underlying pathophysiology of CECs, which is presumably greater in inflammatory than in non-inflammatory vascular diseases. This observation might be explained by the fact that a vasculitis-associated immune cell infiltration of the vessel wall results in a profound destruction of the entire vascular wall including the endothelial cell layer (24). In contrast, histological samples from patients with RCVS do not show quite a destructive process of the vessel wall (8, 9). However, a pathological process of the endothelium also occurs in RCVS. An endothelial thickening but normal remaining wall structures without evidence of an arterial inflammation were observed. Of note, in MMD autopsy also revealed thickening of the intima with an elevated number of smooth muscle cells and an attenuation of the media in affected vessels again without any evidence of an inflammation or an extensive endothelial destruction (7). Together with our and other previous results these findings indicate that CECs may describe the extent of an endothelial injury and, thus, might help to distinguish an active PACNS from RCVS and MMD. Furthermore, based on these results we subsumed both non-inflammatory disease entities into one group within the study cohort.

It may be argued that the increased CEC numbers in the patients with RCVS and MMD might have originated from cerebral ischemic or hemorrhagic events that most of the patients suffered from at the time of CEC measurement. However, others and we have previously shown that CEC levels in patients with ischemic stroke show less CEC numbers than patients with active PACNS and only a small increase of CEC values compared to healthy controls (23). Some limitations may also be argued regarding the patients in our study who received medication at the time of blood sampling that had potentially influenced the CEC numbers. There are several reports on drug therapies, such as aspirin (25), clopidogrel (26), or statins (27), that might reduce CEC numbers, indicating an attenuated vascular injury and potential protective effect on endothelium. However, the number of patients treated by these agents was either low in our patient groups, e.g., clopidogrel, or were even high in active PACNS and RCVS/MMD patients, e.g., statins and aspirin, so that a significant treatment effect on CEC levels was not assumed. However, future prospective studies are necessary to test the effect of medication on CEC numbers.

One drawback of our study is the small number of patients in each group of the study sample. Both, PACNS and its mimics, are rare diseases, so that recruiting a high amount of patients in each subgroup is hampered and remains challenging. Furthermore, our study lacks a previous power calculation. However, required values, such as standard deviations, which may be derived from previous studies on CEC numbers in PACNS compared to RCVS patients, were not available, since such studies have not yet been reported so far. Despite the lack of power calculations and small sample sizes, we demonstrated significant results in the between-group comparisons, even after adjusting for multiple comparisons. Moreover, it was not possible to draw the blood at identical time points after symptom onset in each individual. However, CEC assessment was rather performed at specific time periods, in particular, when PACNS patients and patients with RCVS and MMD were in an active course of disease. Given that there is currently no evidence that CEC numbers may change significantly during an active disease episode, we are confident that this does not diminish our findings and the results are still reliable. In addition, the small group of patients with non-inflammatory vasculopathies consists of two different disease entities which makes this group heterogeneous. Given that both brain vessel diseases show similar vascular alterations and lack vessel inflammation in histopathology, we feel confident to include them into one patient group.

Another potential shortcoming is the relative low rate of PACNS patients with histological evidence (56%, 12 out of 21). Of note, the rate in the two largest patient cohorts published to date, the French and the American cohort, was even lower (both 29%) (21, 28). These numbers in our and previous studies again highlight the difficulties in diagnosing PACNS and emphasize the necessity to identify novel diagnostic tools to increase the diagnostic accuracy.

## Conclusions

CECs are markers for endothelial injury. This study provides preliminary data that an active PACNS leads to increased numbers of CECs in the peripheral blood while CECs are low during clinical remission. Our present study confirmed previous findings indicating CECs as potential diagnostic biomarkers in PACNS and, moreover, CECs as potential biomarkers to monitor treatment. In addition, this study showed that CECs may help to separate PACNS from important differential diagnosis. However, though these findings are very promising, further observational studies are necessary, in particular, in a large cohort of active biopsy-proven vasculitis patients, to draw definitive conclusions.

## Data Availability Statement

The raw data supporting the conclusions of this article will be made available by the authors, without undue reservation, to any qualified researcher.

## Ethics Statement

This study was approved by the local ethics committee (Hamburg, Germany; PV5340). Written informed consent was obtained from all participants or proxies.

## Author Contributions

MD-C: substantial contributions to the conception and design of the work, acquisition, analysis, and interpretation of data for the work, drafting the work, and revising it critically for important intellectual content. HP: analysis of data for the work and revising the work critically for important intellectual content. YD: acquisition of data for the work and revising the work critically for important intellectual content. VH and BR: interpretation of data for the work and revising the work critically for important intellectual content. CG and GT: analysis and interpretation of data for the work and drafting the work and revising it critically for important intellectual content. TM: substantial contributions to the conception of the work, analysis and interpretation of data for the work, drafting the work, and revising it critically for important intellectual content. All authors provide approval for publication of the content and agree to be accountable for all aspects of the work in ensuring that questions related to the accuracy or integrity of any part of the work are appropriately investigated and resolved.

### Conflict of Interest

CG serves on scientific advisory boards for Bayer Vital, Boehringer Ingelheim, Acticor Biotech, Amgen, and Prediction Biosciences; has received funding for travel and/or speaker/consulting honoraria from Bayer Vital, Boehringer Ingelheim, Sanofi Aventis, Amgen, EBS Technologies, GlaxoSmithKline, Lundbeck, Pfizer, Silk Road Medical, and UCB, and Abbott; serves on editorial boards for INFO Neurologie & Psychiatrie and Aktuelle Neurologie and as editor of textbook Therapie und Verlauf neurologischer Erkrankungen; has received grants to supporting employees/scientists of his clinic from Merz Pharmaceuticals, Allergan, Novartis, and NeuroConn; and receives research support from Deutsche Forschungsgesellschaft, the European Union, Wegener Foundation, Schilling Foundation, and Werner-Otto- Foundation. GT has received personal fees as consultant or lecturer from Acandis, Bayer, Boehringer Ingelheim, Bristol-Myers Squibb/Pfizer, Daichi Sankyo, Stryker, and research grants from Bayer, Federal Ministry for Economic Affairs and Energy (BMWi), Corona-Foundation, German Research Foundation (DFG), Else Kröner-Fresenius Foundation, European Union (Horizon 2020), German Innovation Fund. TM has received personal fees from Merck-Serono, Boehringer Ingelheim, Bristol-Myers Squibb/Pfizer, Novartis, Biogen, Grifols, CSL-Behring, grants from the European Union (FP-7, ERA-NET), German Research Foundation, Schilling Foundation, and Werner-Otto Foundation. The remaining authors declare that the research was conducted in the absence of any commercial or financial relationships that could be construed as a potential conflict of interest.
