# Supplementary material for: Circulating Endothelial Cells as Promising Biomarkers in the Differential Diagnosis of Primary Angiitis of the Central Nervous System
Source: Front Neurol. 2020 Mar 31;11:205. doi: 10.3389/fneur.2020.00205 (PMC7137900; doi:10.3389/fneur.2020.00205)
Supplement: Supplementary file 1 [file Data_Sheet_1.docx]

**Supplementary Tables**

**Patients with active PACNS**

| **Pat** | **Age** | **Symptoms** | **MRI/DBI** | **CSF** | **DSA/ MRA** | **Biopsy** | **Disease variant** | **Treatment** |
| --- | --- | --- | --- | --- | --- | --- | --- | --- |
| 1 | 52 | Dementia, gait disturbanceaphasia  (Relapse) | Progressive brain atrophy  DBI: not performed | WBC 14/3 /µl, Pro: 1122 mg/l, OKB: identical bands in the CSF and serum, Cyto: lymphomonocytic pleocytosis | No pathologies | Pos. (LP) | SVV | *Before:* cortisone, CYC, MTX *Now:* RTX |
| 2 | 41 | Headache, left-sided paresthesia  (Disease onset) | Progressive T2-hyperintensities, contrast enhanced lesions  DBI: not performed | WBC 2/3 /µl, Pro: 643mg/l, OCB: neg, Cyto: N.p. | No pathologies | Pos. (LP) | SVV | *Now:* Cortisone, planned immunosuppressive treatment with RTX |
| 3 | 60 | Hemianopsia, cognitive dysfunction  (Disease onset) | Intracranial bleeding, T2-hyperintensities, contrast enhanced lesions, microbleeds  DBI: not perfomed | WBC 4/3 /µl, Pro: 710mg/l, OCB: neg, Cyto: N.p. | No pathologies | Pos.  (ABRA) | SVV | *Now:* Cortisone, CYC |
| 4 | 48 | Aphasia, right-sided hemiplegia  (Relapse) | Multifocal ischemic stroke lesions  DBI: concentric contrast enhanced vessel walls | WBC 4/3 /µl, Pro: 396mg/l, Intrathecal IgA synthesis, OCB: neg, Cyto: N.p. | Multifocal irregular alternating stenosis/ occlusions and dilatations | N.p. | MVV | *Before:* Cortisone, RTX (died rapidly in the following disease course) |
| 5 | 33 | Aphasia, left-sided hemi-paresis  (Relapse) | Multifocal ischemic stroke lesions  DBI: concentric contrast enhanced vessel walls | WBC 4/3 /µl, Pro 379 mg/l, intrathecal IgM synthesis, OCB: neg; Cyto: no pathologies | Progressive multifocal irregular alternating stenosis/ occlusions and dilatations | Neg. | MVV | *Before:*  cortisone, CYC, MTX *Now:* cortisone and RTX |
| 6 | 38 | Aphasia, facial palsy  (Disease onset) | Atypical intracranial hemorrhage, no subarachnoid hemorrhage  DBI: not performed | WBC 16/3 /µl, Pro 516 mg/l, OCB neg., Cyto: N.p. | Multifocal irregular alternating stenosis/occlusions and dilatations, no aneurysm or arterio-venous malfor-mation | N.p. | MVV | Planned immuno-sup-pressive treatment, but lost to follow up |
| 7 | 59 | Dysarthria, right-sided hemi-paresis, headache  (Disease onset) | Multifocal ischemic stroke lesions, contrast enhanced T2-lesions  DBI: concentric contrast enhanced vessel walls | WBC 32/3 /µl, Pro 400mg/l, intrathecal IgM and IgA synthesis, OCB: neg., Cyto: lympho-monocytic pleocytosis | Progressive multifocal irregular alternating stenosis/  occlusions and dilatations, no aneurysm | N.p. | MVV | *Now:* cortisone, CYC |

*Abbreviations: MRI, magnetic resonance imaging; DBI, dark blood imaging; CSF, cerebrospinal fluid; MRA, MR angiography; DSA, digital subtraction angiography; WBC, white blood cell count; Pro, protein level in the CSF; OCB, oligoclonal bands; Cyto, Cytology; LP, lymphocytic pattern; Pos, positive; Neg, negative; N.p., Not performed; SVV, small vessel variant; MVV, medium vessel variant; CYC, cyclophosphamide; RTX, rituximab; MTX, methrothrexate; w.p., without pathologies*

**Patients with PACNS in remission**

| **Pat** | **Age** | **Symptoms** | **MRI/DBI** | **CSF** | **DSA/ MRA** | **biopsy** | **Disease variant** | **Treatment** |
| --- | --- | --- | --- | --- | --- | --- | --- | --- |
| 1 | 52 | *Before:* Encephalo-pathy, headache, seizures  *Now:* no residual clinical symptoms | T2-hyper-intensities, contrast enhanced lesions DBI: not performed | WBC 10/3 /µl, Pro 798 mg/l, intrathecal IgG synthesis, OCB: isolated bands in the CSF; Cyto: no pathologies | No pathologies | Pos. (LP) | SVV | *Before;* cortisone, CYC  *Now:* MTX |
| 2 | 45 | *Before:* Headache, gait disturbance hemianopsia, seizures  *Now:* Residual hemianopsia, clinically stabilized | T2- hyper-intensities, contrast enhanced lesions, microbleeds DBI: no pathologies | WBC 87/3 /ul, Pro 944 mg/l, OCB: neg, Cyto: lympho-monocytic pleocytosis | No pathologies | Pos. (GP) | SVV | *Before:* cortisone, CYC, RTX, MTX *Now:* cortisone, TCZ, MTX |
| 3 | 49 | *Before:* Headache, dizziness, left-sided hemiparesis *Now:* residual left-sided clumsy hand syndrome, spasticity of the left leg, clinically stabilized | Recurrent stroke  DBI: no pathologies | WBC 23/3 /µl, Pro 509 mg/l, OCB: neg, Cyto: lymphocytic pleocytosis, signs of activation | Multifocal irregular alternating stenosis/ occlusions and dilatations | Pos. (LP) | MVV | *Before:* cortisone, CYC, MTX  *Now:* RTX |
| 4 | 57 | *Before:* Headache, encephalo-pathy, dysarthria, seizures *Now:* residual aphasia, dysarthria, right-sided hemiparesis, seizures,  clinically stabilized | T2-hyper-intesities, contrast enhanced lesions  DBI: not performed | WBC 5/3 /µl, Pro 564mg/l, OCB: neg, Cyto: no pathologies | No pathologies | Pos. (LP) | SVV | *Before:* cortisone, CYC, Aza *Now:* medication already withdrawn after 2 years |
| 5 | 67 | *Before:* hemiparesis, vision disturbance  *Now:* residual spasticity and paresis on the left side, intermittent vision disturbance  clinically stabilized | Recurrent ischemic and hemorrhagic stroke  DBI: concentric contrast enhanced vessel walls | *No detailed information:*  No pathologies | Multifocal irregular alternating stenosis/ occlusions and dilatations | Pos. (LP) | MVV | *Before:* cortisone, CYC, Aza *Now:* oral cortisone, RTX |
| 6 | 56 | *Before:* headache, cognitive decline, vision disturbance  *Now:* residual slight headache and mnestic deficits, improved after medication,  clinically stabilized | T2-hyper-intesities  DBI: not performed | WBC 3/3 /µl, Pro 276 mg/l, OCB neg.; Cyto: N.p. | No pathologies | Pos. (ABRA) | SVV | *Before:* cortisone  *Now:* Aza |
| 7 | 32 | *Before:* right-sided hemiparesis  *Now:*  no residual clinical symptoms | Recurrent ischemic stroke DBI: not performed | *Lumber puncture performed in external hospital, no detailed information:*  no pathologies | Multifocal irregular alternating stenosis/ occlusions and dilatations | *Biopsy performed in external hospital, no detailed information:* Pos. | MVV | *Before:* cortisone, CYC, MMF  *Now:*  medication already withdrawn after 2 years |
| 8 | 39 | *Before:* seizures, dizziness, headache *Now:*  no residual clinical symptoms | T2-hyper-intesities, contrast enhanced lesions  DBI: no pathologies | WBC 6/3 /µl, Pro 445 mg/l, OCB: isolated bands in the CSF, Cyto: N.p. | Multifocal irregular alternating stenosis/ occlusions and dilatations | Pos. (GP) | MVV | *Before:* cortisone, RTX, MTX,  *Now:* MTX, IFX |
| 9 | 71 | *Before:*  headache, hemianopsia *Now*: no residual clinical symptoms | T2-hyper-intesities, contrast enhanced lesions  DBI: concentric contrast enhanced vessel walls | WBC 15/3 /µl, Pro: 502 mg/l, OCB: identical bands in the CSF and serum, Cyto: lymphocytic pleocytosis | Multifocal irregular alternating stenosis/ occlusions and dilatations | Pos (NP) | MVV | *Before:* Cortisone, CYC, MTX  *Now:*  medication already withdrawn after 2 years |
| 10 | 46 | *Before:*  Headache, encephalo-pathy, depression  *Now:*  no residual clinical symptoms | Multifocal T2-hyper-intensities, contrast enhanced lesions DBI: not performed | WBC 103/3 /µl, Pro 592 mg/l, intrathecal IgA and IgM synthesis, OCB: pos., Cyto: lympho-monocytic pleocytosis | No pathologies | Pos (GP/LP) | SVV | *Now:*  Cortisone, CYC |
| 11 | 84 | *Before:*  Seizures, apraxia, aphasia, cognitive dysfunction  Now:  Residual gait disturbance, clinically stabilized | Multifocal T2-hyper-intensities, contrast enhanced lesions DBI: not performed | WBC 29/3 /µl, Pro 1138 mg/l, intrathecal IgA and IgM synthesis, OCB: neg., Cyto: lymphocytic pleocytosis | No pathologies | Pos. (ABRA) | SVV | *Before:*  Cortisone, CYC  *Now:* Aza |
| 12 | 57 | *Before:*  Seizures, cognitive dysfunctions  *Now:*  No residual clinical symptoms | Multifocal T2-hyper-intensities  DBI: not performed | WBC 4/3 /µl, Pro 1013 mg/l, OCB: identical bands in the CSF and serum, Cyto: no pathologies | No pathologies | Pos. (ABRA) | SVV | *Now:* Cortisone, CYC |
| 13 | 33 | *Before:* internuclear ophthalmo-plegia  *Now:*  No residual clinical symptoms | Recurrent ischemic stroke  DBI: concentric contrast enhanced vessel walls | WBC 7/3 /µl, Pro 358 mg/l, OCB: identical bands in the CSF and serum, Cyto: no pathologies | Multifocal irregular alternating stenosis/ occlusions and dilatations | N.p. | MVV | *Before:* cortisone  *Now:* RTX |
| 14 | 72 | *Before:* right-sided hemiparesis, dysarthria  *Now:* residual paresis of the right hand, dysarthria, clinically stabilized | Recurrent ischemic and hemorrhagic stroke  DBI: concentric contrast enhanced vessel walls | WBC 4/3 /µl, Pro 431 mg/l, OCB: identical bands in the CSF and serum, Cyto: no pathologies | Multifocal irregular alternating stenosis/ occlusions and dilatations | Neg. | MVV | *Before:* cortisone, CYC  *Now:*  no mainte-nance therapy (patient refused to take MMF) |
| 15 | 61 | *Before:* headache, dysarthria, left-sided, hemiparesis, tremor, cognitive decline  *Now:* residual cognitive deficits, depression, tremor, left-sided paresthesia, clinically stabilized | Recurrent ischemic stroke, T2-hyper-intensities, contrast enhanced lesions  DBI: concentric contrast enhanced vessel walls | WBC 859/3 /µl, Pro 942mg/l, intrathecal IgM synthesis, OCB: identical bands in the CSF and serum, Cyto: lymphocytic pleocytosis, signs of activation | Multifocal irregular alternating stenosis/ occlusions and dilatations | N.p. | MVV | *Before:* cortisone, CYC, MTX  *Now:* MTX |
| 16 | 37 | *Before:*  Hemianopsia Headache  *Now:*  Residual hemianopsia,  clinically stabilized | Ischemic stroke DBI: concentric contrast enhanced vessel walls | WBC 0/3 /µl, Pro 247 mg/l, OCB: pos, Cyt: N.p. | Multifocal irregular stenosis and dilatations | N.p. | MVV | *Before:*  Cortisone, RTX  *Now:* RTX |
| 17 | 27 | *Before:*  Headache, recurrent left-sided paresis  Now:  Intermittent headache, clinically stabilized | Recurrent ischemic stroke  DBI:  concentric contrast enhanced vessel walls | WBC 64/3 /µl, Pro 608 mg/l, OCB: neg. Cyto: lympho-monocytic pleocytosis, signs of activation | Multifocal irregular alternating stenosis/ occlusions and dilatations | N.p. | MVV | *Now:*  Cortisone, RTX |
| 18 | 56 | *Before:*  Right-sided hemiparesis, dysarthria, hemianopsia  *Now:*  Residual hemiparesis, clinically stabilized | Recurrent ischemic stroke  DBI: not performed | WBC 2/3 /µl, Pro 242 mg/l, OCB: identical bands in the CSF and serum, Cyto: no pathologies | Multifocal irregular alternating stenosis/ occlusions and dilatations | N.p. | MVV | *Before:*  Cortisone, CYC  *Now:* MTX |
| 19 | 39 | *Before:*  Right-sided hemiparesis, aphasia, left-sided hemiparesis  *Now:*  Residual tetraparesis, global aphasia, clinically stabilized | Intracranial bleeding, recurrent ischemic stroke  DBI: neg. | WBC 30/3 /µl, Pro 1081 mg/l, OCB: identical bands in the CSF and serum, Cyto: lymphomoncytic pleocytosis with also granulocytes | Progressivemultifocal irregular alternating stenosis/ occlusions and dilatations | N.p. | MVV | *Now:*  Cortisone  (further immunosuppressive treatment planned) |
| 20 | 45 | *Before:*  Left-sided hemiparesis  *Now:*  No residual clinical symptoms | T2-hyper-intensities, contrast enhanced lesions, recurrent ischemic stroke  DBI: concentric contrast enhanced vessel walls | WBC 43/3 /µl, Pro 226 mg/l, OCB: isolated bands in the CSF, intrathecal IgG, IgM, IgA synthesis Cyto: lymphomoncytic pleocytosis | Irregular alternating stenosis/ occlusions and dilatations | Neg. | MVV | *Now:*  Cortisone, CYC |

*Abbreviations: MRI, magnetic resonance imaging; DBI, dark blood imaging; CSF, cerebrospinal fluid; MRA, MR angiography; DSA, digital subtraction angiography; WBC, white blood cell count; Pro, protein level in the CSF; OCB, oligocloncal bands; Cyto, Cytology; Pos, positive; Neg, negative; SVV, small vessel variant; MVV, medium vessel variant; CYC, cyclophosphamide; RTX, rituximab; MTX, methrothrexate; Aza, azathioprine; TCZ, tocilizumab; IFX, infliximab; MMF, mycophenolate mofetil; LP, lymphocytic pattern; G, granulomatous pattern; NP, necrotizing pattern; ABRA, amyloid-beta related angiitis*

**Patients with MMD and RCVS**

| **Pat** | **Age** | **Diagnosis** | **Symptoms** | **MRI/DBI** | **CSF** | **DSA/ MRA** | **Comments** |
| --- | --- | --- | --- | --- | --- | --- | --- |
| 1 | 45 | MMD | *Before:* residual dysarthria, left-sided clumsy hand *Now:* progressive headache resistant to medication, progressive aneurysm | Recurrent ischemic stroke  DBI: no pathologies | WBC 3/3 /ul, Pro 512 mg/l, OCB: neg, Cyto: no pathologies | ICA and MCA occlusion on the right side, stenosis on the left side, moyamoya syndrome | STA-MCA Bypass in the past |
| 2 | 57 | MMD | Acute aphasia, dysarthria | Intracranial hemor-rhage DBI: no pathologies | WBC 5/3 /ul, Pro 451 mg/l, OCB: neg, Cyto: N.p. | ACA and MCA occlusion on the left side, moyamoya syndrome | STA-MCA Bypass discussed |
| 3 | 23 | MMD | *Before:*  Headache, left-sided hemiparesis, hemianopsia  *Now:* aphasia, dysarthria, residual hemianopsia | Recurrent ischemic stroke, intracranial bleeding  DBI: not performed | WBC 7/3 /ul, Pro 304 mg/l, OCB: neg, Cyto: N.p. | Progressive ICA and MCA occlusion on the left and right side, moyamoya syndrome on both sides | STA-MCA Bypass discussed |
| 4 | 29 | RCVS | TCH after a head trauma, recurrent TCH *Later:*  no clinical symptoms | No pathologies DBI: no pathologies | WBC 1/3 /ul, Pro 399 mg/l OCB neg, Cyto: N.p. | MCA occlusion on the left side | MRA follow up imaging:  4 months, persistent vascular abnormality, 2^nd^ follow up brain imaging planned |
| 5 | 51 | RCVS | Postcoital TCH, recurrent TCH *Later:* no clinical symptoms | No pathologies DBI: not performed | WBC 1/3 /ul, Pro 638 mg/l, OCB: not assessed, Cyto; no pathologies | “String and bead”- appearance of the cerebral arteries | MRA follow up imaging:  4 months, mostly resolved vascular changes |
| 6 | 45 | RCVS | Severe headache resistant to medication, vision disturbance, left-sided paresthesia *Later:*  no clinical symptoms | Ischemic stroke, minor subarach-noidal hemor-rhage | WBC 3/3 /ul, Pro 391 mg/l, OCB: neg, Cyto: no pathologies | “String and bead”- appearance of the cerebral arteries, no aneurysm, resolved vessel alterations after intraarterial application of nimodipine | MRA follow up imaging:  2 months, mostly resolved vascular abnormalities |
| 7 | 30 | RCVS | Peri-/postpartum severe headache, left-sided sensory loss, visual disturbance | No pathologies DBI: not performed | Not performed | “String and bead”- appearance of the cerebral arteries | MRA follow up imaging:  4 months, resolved vascular changes |

*Abbreviations: MRI, magnetic resonance imaging; DBI, dark blood imaging; CSF, cerebrospinal fluid; MRA, MR angiography; DSA, digital subtraction angiography; MMD, moyamoya disease; RCVS,* reversible cerebral vasoconstriction syndrome; STA, superficial temporal artery; MCA, middle cerebral artery; ACA, anterior cerebral artery; *WBC, white blood cell count; Pro, protein level in the CSF; OCB, oligocloncal bands; Cyto, Cytology; TCH, thunderclap headache*
