# Supplementary material for: Circulating Endothelial Cells as Promising Biomarkers in the Differential Diagnosis of Primary Angiitis of the Central Nervous System
Source: Front Neurol. 2020 Mar 31;11:205. doi: 10.3389/fneur.2020.00205 (PMC7137900; doi:10.3389/fneur.2020.00205)
Supplement: Supplementary file 2 [file Data_Sheet_2.docx]

**Supplementary information**

**Patients with probable PACNS**

Patients were considered as probable PACNS when other differential diagnosis such as infectious diseases, non-inflammatory vasculopathies, systemic vasculitis or malignancies had been ruled out and if neurological symptoms were indicative of PACNS (e.g. chronic progressive headache, encephalopathy, cognitive decline, seizures), cranial magnetic resonance imaging (MRI) scans including dark blood imaging (DBI) sequences and angiography (digital subtraction angiography and/or MR angiography (MRA)) were compatible with PACNS, cerebrospinal fluid (CSF) analysis were abnormal, an ultrasound of the brain supplying vessels ruled out a germane arteriosclerosis and immune suppressive therapy was started (or planned to be initiated).

**Patients with RCVS and MMD**

Given that validated diagnostic criteria of RCVS are lacking_[1]_, the diagnosis of RCVS was adapted from the International Classification of Headache disorder, 3^rd^ edition, and experienced-based guidelines, proposed by Calabrese et al. earlier_[2]_. The diagnosis included severe or acute (thunderclap) headache, normal or near-normal CSF analyses (if performed), proven vessel irregularities, e.g. “string and beads”-patterns, in the conventional angiography without evidence of an aneurysmal subarachnoid hemorrhage (SAH). In addition, based on recent studies a significant response of brain vessel irregularities to an intra-arterial application of nimodipine was considered to be a strong diagnostic and treatment tool in RCVS suspected patients of our study_[3,4]_. Furthermore, persistent vessel abnormalities in follow up imaging were no reason to suspect another diagnosis, given that persistent vessel alterations are in line with the findings of the largest case series of 139 patients with RCVS to date, in which 24% showed only partially resolved arterial abnormalities in follow-up images_[5,6]_.
The diagnosis of MMD was made referring to the revised diagnostic criteria of the Research Committee of MMD of the Japanese Ministry of Health, Labour and Welfare (2015) that include patients with both bilateral and unilateral steno-occlusive changes of the distal carotid artery and/or the proximal part of the anterior/middle cerebral artery, and with abnormal vascular network at the base of the brain_[7,8]_.

**Detailed information on CEC measurement**

Since previous studies demonstrated that manipulation with the needle at site of venipuncture may result in a dislodgment of endothelial cells_[9]_, withdrawal from permanent (central) venous lines was avoided and the first tube after withdrawal of the peripheral blood was discarded to avoid false-positive CEC numbers. Previous studies on CEC measurement in ANCA-positive vasculitis patients revealed a significant reduction of CEC numbers at room temperature (25%) and at 4°C (12.5%) after storage for 24 hours_[10]_. Therefore, we performed the CEC assessment at 4°C immediately after withdrawal instead of storage to avoid any bias.

**Patients with PACNS in remission**

For initial treatment most of the patients received prednisone therapy followed by cyclophosphamide and/or rituximab medication. Two patients required further escalation of the therapy with tocilizumab and infliximab. Maintenance therapy was given to keep the disease under control and limit the risk of relapses. In most of the cases methotrexate or azathioprine, in two cases mycophenolate mofetil, were given. In three cases the maintenance therapy was already withdrawn after two years of treatment at the time of CEC assessment, another patient had refused to take further medication after induction treatment.

***References***

1. Singhal AB, Hajj-Ali RA, Topcuoglu MA, et al. Reversible cerebral vasoconstriction syndromes: analysis of 139 cases. *Arch Neurol*. 2011; 68: 1005-1012.
2. Calabrese LH, Dodick DW, Schwedt TJ and Singhal AB. Narrative review: reversible cerebral vasoconstriction syndromes. *Ann Intern Med*. 2007; 146: 34-44.
3. Linn, J., Fesl, G., Ottomeyer, C., Straube, A., Dichgans, M., Bruckmann, H., et al. (2011). Intra-arterial application of nimodipine in reversible cerebral vasoconstriction syndrome: a diagnostic tool in select cases? *Cephalalgia* 31(10)**,** 1074-1081. doi: 10.1177/0333102410394673.
4. Kraayvanger, L., Berlit, P., Albrecht, P., Hartung, H.P., and Kraemer, M. (2018). Cerebrospinal fluid findings in reversible cerebral vasoconstriction syndrome: a way to differentiate from cerebral vasculitis? *Clin Exp Immunol* 193(3)**,** 341-345. doi: 10.1111/cei.13148.
5. Calabrese, L.H., Dodick, D.W., Schwedt, T.J., and Singhal, A.B. (2007). Narrative review: reversible cerebral vasoconstriction syndromes. *Ann Intern Med* 146(1)**,** 34-44.
6. Singhal, A.B., Hajj-Ali, R.A., Topcuoglu, M.A., Fok, J., Bena, J., Yang, D., et al. (2011). Reversible cerebral vasoconstriction syndromes: analysis of 139 cases. *Arch Neurol* 68(8)**,** 1005-1012. doi: 10.1001/archneurol.2011.68.
7. Fujimura M, Bang OY and Kim JS. Moyamoya Disease. *Front Neurol Neurosci*. 2016; 40: 204-220.
8. Hishikawa T, Sugiu K and Date I. Moyamoya Disease: A Review of Clinical Research. *Acta Med Okayama*. 2016; 70: 229-236.
9. Woywodt A, Streiber F, de Groot K, Regelsberger H, Haller H, Haubitz M.Circulating endothelial cells as markers for ANCA-associated small-vessel vasculitis.Lancet. 2003 Jan 18;361(9353):206-10.
10. Woywodt A, Blann AD, Kirsch T, Erdbruegger U, Banzet N, Haubitz M, Dignat-George F. Isolation and enumeration of circulating endothelial cells by immunomagnetic isolation: proposal of a definition and a consensus protocol.J Thromb Haemost. 2006 Mar;4(3):671-7.
